# The influence of systemic inflammation, dietary intake and stage of disease on rate of weight loss in patients with gastro-oesophageal cancer

**DOI:** 10.1038/sj.bjc.6604828

**Published:** 2009-01-06

**Authors:** D A C Deans, B H Tan, S J Wigmore, J A Ross, A C de Beaux, S Paterson-Brown, K C H Fearon

**Affiliations:** 1 University Department of Surgery, Royal Infirmary, 51 Little France Crescent, Old Dalkeith Road, Edinburgh EH16 4SA, UK

**Keywords:** inflammation, cachexia, SIMS

## Abstract

Although weight loss is often a dominant symptom in patients with upper gastrointestinal malignancy, there is a lack of objective evidence describing changes in nutritional status and potential associations between weight loss, food intake, markers of systemic inflammation and stage of disease in such patients. Two hundred and twenty patients diagnosed with gastric/oesophageal cancer were studied. Patients underwent nutritional assessment consisting of calculation of body mass index, measurement of weight loss, dysphagia scoring and estimation of dietary intake. Serum acute-phase protein concentrations were determined by enzyme-linked immunosorbent assay. In all, 182 (83%) patients had lost weight at diagnosis (median loss, 7% body weight). Weight loss was associated with poor performance status, advanced disease stage, dysphagia, reduced dietary intake and elevated serum C-reactive protein (CRP) concentrations. Multiple regression identified dietary intake (estimate of effect, 38%), serum CRP concentrations (estimate of effect, 34%) and stage of disease (estimate of effect, 28%) as independent variables in determining degree of weight loss. Mechanisms other than reduced dietary intake or mechanical obstruction by the tumour appear to be involved in the nutritional decline in patients with gastro-oesophageal malignancy. Recognition that systemic inflammation plays a role in nutritional depletion may inform the development of appropriate therapeutic strategies to ameliorate weight loss, making patients more tolerant of cancer-modifying treatments such as chemotherapy.

Cachexia remains an important cause of morbidity and mortality among cancer patients, affecting up to 85% of patients with gastrointestinal malignancy at the time of diagnosis (Alexandrakis *et al*, 2003). Cancer cachexia is associated with reduced quality of life scores, reduced performance status, lower response rates to chemotherapy and overall poorer outcomes (DeWys *et al*, 1980; Ross *et al*, 2004; Barber *et al*, 1999a). Around 20% of deaths from cancer may be directly related to cachexia (Studley, 1936; Inagaki *et al*, 1974). In a large study of patients with oesophageal cancer, weight loss greater than 10% pre-morbid weight was the only significant predictor of early death in patients undergoing surgical resection (Kelson *et al*, 1998).

The weight loss that is commonly associated with gastro-oesophageal malignancy is generally assumed to be secondary to the mechanical effects of the tumour on the upper digestive tract. Obstruction to swallowing, early satiety, nausea and vomiting are common symptoms associated with these tumours. However, some of these patients may remain relatively weight stable whereas other patients show marked and rapid weight loss despite apparent similarities in the degree of dysphagia and stage of disease. In addition, weight loss and cachexia are commonly associated with types of cancer not directly involving the gastrointestinal tract, such as non-small cell lung cancer (Scott *et al*, 2002). A possible explanation for these variations in nutritional status may relate to the co-existence of systemic inflammation. Up to 50% of patients with cancer have an acute-phase protein response (APPR) at the time of diagnosis, including patients with upper gastrointestinal malignancy (Falconer *et al*, 1994, 1995; Chen *et al*, 1999; Barber *et al*, 1999b; Alexandrakis *et al*, 2003). Elevated acute-phase protein concentrations have earlier been linked with increased weight loss among patients with cancer. Elevated serum C-reactive protein (CRP) concentrations have been correlated positively with weight loss in human cancer patients (Falconer *et al*, 1995; Wigmore *et al*, 1997; O'Gorman *et al*, 1999). An APPR in pancreatic cancer patients is associated with elevated resting-energy expenditure (REE) and reduced energy intake (Falconer *et al*, 1994; Wigmore *et al*, 1997). In patients with cancer, therefore, an APPR has been associated with hypermetabolism, anorexia, increased weight loss and adverse prognosis (Argiles *et al*, 1997).

The clinical sequelae of systemic inflammation include alterations in protein and fat metabolism, changes in energy expenditure and alterations in patterns of hormonal secretion (Selby *et al*, 1987; Baumann and Gauldie, 1994; Stouthard *et al*, 1995; Baracos *et al*, 2003). These metabolic changes may underpin the syndrome of cachexia and may in part be mediated through increased pro-inflammatory cytokine activity. Circulating concentrations of IL-1*β*, IL-6 and TNF*α* and other cytokines have been associated with anorexia and weight loss in rodent tumour models and in human participants (Gelin *et al*, 1991; Strassmann *et al*, 1992). More recently, IL-6 overexpression in the serum and in tumour specimens of pancreatic cancer patients has been linked with accelerated weight loss (Martignoni *et al*, 2005). Therefore, in patients with cancer, the underlying metabolic alterations associated with systemic inflammation may contribute to the development of weight loss and cachexia in these patients.

This study describes the changes in nutritional status in a cohort of patients with gastro-oesophageal cancer at the time of diagnosis and investigates the contribution of systemic inflammation to their nutritional decline.

## Materials and methods

### Study population

All patients diagnosed with gastric or oesophageal cancer within the Lothian and Borders regions between March 2002 and June 2004 were eligible for inclusion into the study. No patients were excluded and patients were recruited to the study within 2 weeks of the time of diagnosis. All participants provided written informed consent and the study received ethical permission from the Lothian Research Ethics Committee. Every patient underwent an assessment of his/her nutritional status at recruitment and blood was collected at the same time for determination of serum acute-phase protein concentrations. Patients were staged and underwent various treatments following discussion at the multidisciplinary team meeting. Duration of survival, defined from time of histological diagnosis to death, was recorded for each patient.

### Nutritional assessment

All patients underwent an assessment of their nutritional status at the time of diagnosis. This involved calculation of body mass index (BMI) and estimation of weight loss. Dietary intake was estimated by patients and was validated in a subgroup of patients using detailed food diaries. The severity of dysphagia was also assessed and documented.

### Calculation of BMI and rate of weight loss

Patients were evaluated for height with a stadiometre and weight on spring balance scales, wearing light clothing, without shoes. Patient recall, verified if possible from medical records, was used to determine pre-morbid weight. Pre-morbid weight was recorded in the medical case notes for 103 (47%) patients who had attended an outpatient appointment and had been weighed in the previous 12 months. Body mass index and percentage weight loss compared with pre-morbid weight were calculated. Rate of weight loss was defined as the total amount of weight lost by the patient divided by the number of months of symptoms experienced by the patient. This was chosen as the arbitrary definition of rate of weight loss to gain some information on the rate of weight loss for each patient.

### Estimation of dietary intake and assessment of dysphagia

Dietary intake was estimated by all patients and scored as 1=normal, 2=reduced and 3=poor/minimal. This simple assessment of dietary intake was validated in a subgroup of patients using detailed food diaries. Patients (*n*=22) were instructed to record all food and drink consumed over a 3-day period, which included a weekend day, and the data were analysed by a senior dietician who provided information on the level of intake of macronutrients using computer software (CompEat®, Nutrition Systems, Grantham, UK). Comparisons were made to dietary reference values issued by the Department of Health (1991).

The severity of dysphagia was assessed by interview and was scored according to Knyrim *et al* (1993).

### Determination of serum acute-phase protein concentrations

Blood was collected from every patient at the time of diagnosis and before any therapeutic intervention. All patients were free from infection at the time of blood collection as determined by clinical assessment. C-reactive protein, haptoglobin, *α*_1_-antichymotrypsin (ACT), albumin and transferrin were selected as the acute-phase proteins for investigation. C-reactive protein, haptoglobin and ACT represent positive acute-phase proteins and albumin and transferrin represent negative acute-phase reactants.

Serum CRP concentration was measured using an automated immunoturbidimetric assay within the Clinical Biochemistry Department, Edinburgh Royal Infirmary, UK. This method is a high-sensitivity assay with a lower limit of detection of 0.2 mg l^−1^. Using this assay, a concentration of 10 mg l^−1^ represents the upper limit of the normal range, with most healthy individuals having a serum concentration <2 mg l^−1^ (Gabay and Kushner, 1999). The coefficient of variation was less than 3%. Serum albumin concentration was measured by the automated bromocresol green dye-binding technique also in the Clinical Biochemistry Department. Normal serum concentrations are 35–50 g l^−1^. The coefficient of variation was less than 3%.

Serum concentrations of haptoglobin, ACT and transferrin were determined by sandwich enzyme-linked immunosorbent assay as described earlier (Wigmore *et al*, 2002). The lower limit of sensitivity for each assay was 100 pg ml^−1^ for haptoglobin (coefficient of variation <10%), 50 pg ml^−1^ for ACT (coefficient of variation <10%) and 30 pg ml^−1^ for transferrin (coefficient of variation <10%).

### Statistical analysis

Non-parametric analysis was performed in all instances. Correlations were investigated by Spearman's rank test. Independent variables were analysed by the Mann–Whitney *U-* or the Kruskal–Wallis test. Categorical data were analysed by the *χ*^2^-test. Receiver operator characteristic (ROC) curves were used to evaluate the ability of serum acute-phase protein concentrations to identify patients with the fastest rate of weight loss. Multiple regression modelling was used to identify the variables that were independently associated with weight loss and to calculate the estimates of size of effect.

## Results

### Study patients

Group demographics are presented in Table 1. In summary, 220 patients were studied over a 28-month period. Ninety-five (43%) patients underwent surgical resection and 25 of these received pre-operative chemotherapy. In all, 7 (3%) patients received chemoirradiation with curative intent. The remaining 118 patients (54%) were not suitable for curative therapy and received palliative treatment, such as chemotherapy or endoscopic stenting. One-third (34%) of patients had metastatic disease (stage IV) at the time of presentation and most other patients (*n*=86; 39%) had locally advanced (stage III) disease at diagnosis. About one-quarter (*n*=59; 27%) of patients had early-stage disease (stage I/II).

Patients were followed up for an average of 32 months and minimum of 18 months (range, 18–45 months). At the time of censoring the data, 147 (67%) patients had died. Overall median survival was 13 months.

### Assessment of nutritional status

#### Weight loss

The nutritional variables for the patient group measured at the time of diagnosis are also shown in Table 1. Patients had lost a median of 7.1% (inter-quartile range, 1.2–14.2%) of their total body weight at the time of diagnosis compared with their pre-morbid stable body weight. This was equivalent to a median rate of weight loss of 2.5% total body weight per month of illness (inter-quartile range, 0.3–6.5% per month) (defined from the onset of symptoms). Only 38 (17%) patients remained weight steady at the time of diagnosis, whereas 85 (39%) patients had lost more than 10% of their body weight.

#### Assessment of dietary intake and dysphagia

Eighty-five (39%) patients described their dietary intake as normal, 103 (47%) patients described their intake as reduced compared with normal and 32 (14%) patients had a poor or minimal food intake at diagnosis. Assessment of dietary intake was validated in a subgroup of patients (*n*=22) using detailed food diaries, where actual food intake was compared with perceived food intake. For this patient subgroup, the median energy intake was 2027 kcal day^−1^ (inter-quartile range, 1415–2228 kcal day^−1^) and the median protein intake was 72 g day^−1^ (range, 58–92 g day^−1^). Absolute values were normalised to the estimated average requirement for energy intake and to the reference nutritional intake for protein intake (Table 1). Patient perception of reduced food intake was associated with reduced total calorie intake and reduced protein intake (*P*=0.040 and 0.003, respectively; Mann–Whitney *U*-test) (data not shown).

Reduced dietary intake was associated with a lower BMI at diagnosis (*P*=0.007, Kruskal–Wallis test), increased total weight loss (*P*<0.001) and increased rate of weight loss (*P*<0.001). In addition, reduced food intake was associated with reduced Karnofsky performance scores (*P*<0.001, Kruskal–Wallis test) and increased dysphagia scores (*P*<0.001, *χ*^2^-test).

Eighty-nine (40%) patients had no dysphagia at diagnosis, 95 (43%) were able to swallow solid or semisolid food, 32 (15%) were able to swallow liquids only and 4 (2%) patients had total dysphagia. Increasing dysphagia was also linked with increased weight loss.

### Relationship between patient clinicopathological characteristics and nutritional status

**Figure 1:**
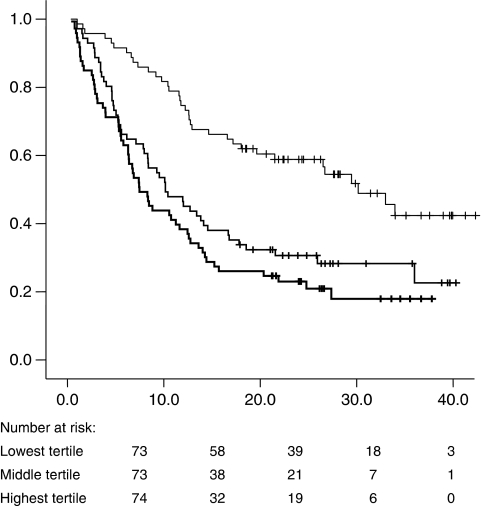
Survival curve representing survival duration in the patient cohort from time of diagnosis stratified according to tertiles of rate of weight loss. Thin line=lowest rate of weight-loss tertile with a median survival of 30.2 months; middle line=middle rate of weight-loss tertile with a median survival of 10.2 months; thick line=highest rate of weight-loss tertile with a median survival of 7.5 months (*P*<0.0001, log-rank test).

Oesophageal tumours were associated with higher dysphagia scores (*P*<0.001, *χ*^2^-test), but not with reduced dietary intake (*P*=0.612) or increased weight loss (*P*=0.320). Total weight loss was similar between the different histological subtypes (*P*=0.206). Advanced disease stage at the time of diagnosis was associated with greater total weight loss (*P*<0.001), increased dysphagia scores (*P*<0.001) and reduced dietary intake (*P*<0.001). Patients with stage III and stage IV disease had lost a median 9.5% (inter-quartile range, 4.3–16.0%) of their total body weight by the time of diagnosis compared with patients with stage I and II disease (median weight loss, 1.4%; inter-quartile range, 0–7.3%). Increased rate of weight loss from the time of diagnosis was associated with adverse prognosis (Figure 1). The median survival for patients in the tertile with the lowest rate of weight loss was 30.2 months; the median survival for those in the intermediate weight-loss tertile was 10.2 months; and the median survival for those patients with the fastest rate of weight loss was 7.5 months (*P*<0.0001, log-rank test).

### Relationship between markers of systemic inflammation and nutritional status

#### Serum acute-phase protein concentrations

The median CRP concentration was 7 mg l^−1^ (inter-quartile range, 2–25 mg l^−1^), and at the time of diagnosis 121 (55%) patients had a CRP concentration greater than 5 mg l^−1^.

#### Serum markers of systemic inflammation and nutritional status

**Figure 2:**
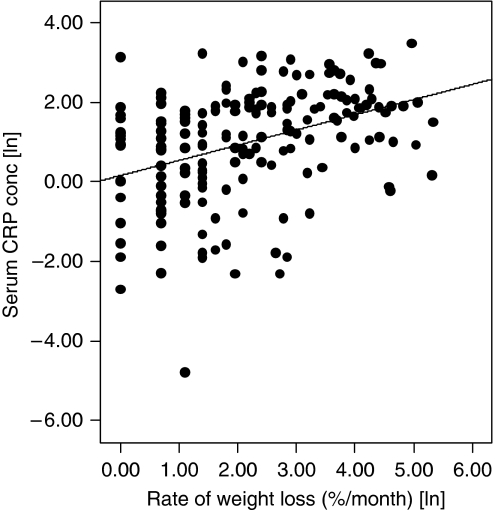
Scatter plot illustrating the positive correlation between elevated serum CRP concentrations and rate of weight loss measured at the time of diagnosis (*P*<0.001, *r*=0.36; Spearman's rank analysis). The *y*-axis represents serum CRP concentrations in mg l^−1^ and the *x-*axis represents the percentage body weight lost per month of symptoms. Given that the data are non-parametric, these values have undergone logarithmic transformation.

Serum acute-phase protein concentrations were associated with nutritional variables. Elevated concentrations of the positive acute-phase proteins (CRP, *α*1-antichymotrypsin and haptoglobin) were associated with increased total weight loss and increased rates of weight loss at the time of diagnosis, whereas the converse was true for the negative acute-phase reactants (albumin and transferrin). Serum CRP concentration was the best predictor of rate of weight loss, as identified by an ROC curve (area under the curve=0.72 (*P*<0.001; 95% confidence interval 0.65–0.79)). The relationship between serum CRP concentration at diagnosis and rate of weight loss is shown in Figure 2.

A cutoff value of 5 mg l^−1^ was chosen to represent an elevated serum CRP concentration. Patients were grouped into tertiles according to the rate of weight loss; lowest tertile median rate of weight loss=0% per month (inter-quartile range, 0–0.3% per month), middle tertile median rate of weight loss=2.4% per month (inter-quartile range, 1.5–3.3% per month) and highest tertile median rate of weight loss=7.6% per month (inter-quartile range, 6.4–14.5% per month). Increasing weight-loss tertile was associated with elevated serum concentrations of CRP (*P*<0.001, *χ*^2^-test) (Table 2).

### Multivariate analysis

Levels of dietary intake, dysphagia score, stage of disease, treatment modality and serum acute-phase protein concentrations (CRP) were all associated with rate of weight loss among the patient group. Multiple regression analysis was performed to identify the variables that were independently associated with weight loss. Levels of dietary intake and dysphagia scores were identified as confounding variables and, therefore, dietary intake was retained in the multivariate analysis and dysphagia scores were excluded. Similarly, stage of disease and treatment modality were also confounding variables, and stage of disease alone was analysed. Dietary intake (*r*=0.28, *P*<0.001), stage of disease (*r*=0.19, *P*=0.003) and serum CRP concentrations (*r*=0.18, *P*=0.007) all retained independent association in determining the extent of weight loss (Table 3). A multivariate general linear model was then used to calculate the estimates of size of effect on degree of weight loss for each variable (Table 3). These data suggested that, overall, 38% of weight loss was determined by level of dietary intake, 34% by serum CRP concentrations (systemic inflammation) and 28% was attributable to stage of disease.

## Discussion

This study has described the changes in nutritional status observed at the time of diagnosis in a cohort of patients with gastro-oesophageal cancer and has investigated the association between these nutritional variables and markers of systemic inflammation. At the time of diagnosis, patients with gastro-oesophageal cancer show a median loss of 7% of their body weight at a rate of 2.5% body weight loss per month. Eighty-three percent of patients had lost weight at the time of diagnosis. Increased weight loss was associated with elevated serum acute-phase protein concentrations. Weight loss was also associated with reduced dietary intake, palliative treatment modalities and advanced stage of disease. Multiple regression analysis identified level of dietary intake (estimate of effect of size, 38%), serum CRP concentrations (estimate of effect of size, 34%) and stage of disease (estimate of effect of size, 28%) as independent variables in determining the degree of weight loss.

Weight loss is common among patients with cancer, particularly among patients with gastrointestinal malignancy in which up to 85% of patients may be affected during their illness (DeWys *et al*, 1980). One hundred and eighty-three (83%) patients in our study had lost weight at the time of diagnosis with almost half of these patients losing 10% or more of their pre-illness body weight. Assuming a linear course, the median rate of weight loss was 2.5% body weight per month (approximately 1.9 kg per month) and compares with previous study relating to patients with pancreatic cancer where the mean rate of weight loss was 2.3 kg per month (Barber *et al*, 1999a; Fearon *et al*, 2003). Pancreatic cancer is notorious for its association with marked weight loss (DeWys *et al*, 1980; Galizia *et al*, 2002).

Almost two-thirds (61%) of patients described their level of dietary intake as reduced or poor compared with their normal levels of intake and, not surprisingly, reduced dietary intake was associated with increased total weight loss and increased rate of weight loss. Recent studies have shown loss of appetite to be an independent prognostic factor for survival in patients with gastro-oesophageal cancer (McKernan *et al*, 2008). In this study, we found that patients' perception of their level of dietary intake reflected actual levels of energy and protein intake as determined from detailed food diaries. Diet diaries have been shown earlier to reflect usual daily food intake, and it would appear that patients' perception of their level of food intake may also reflect actual levels of dietary intake (Bingham *et al*, 1982; Gibson, 1990). We accept that recalled weight loss will be subjected to some error. However, earlier studies have found a good correlation between recalled stable weight and weight recorded in patients' hospital notes before their current illness. A study of Japanese men found that weight recalled by patients strongly correlated with measured weight (*r*=0.849) (Tamakoshi *et al*, 2003). We also accept that the main error in calculating rate of weight loss will come from the duration of weight loss where the calculation is reliant on two items of patient recall. Within these limitations, it is readily possible to discriminate those who are weight stable from those patients who lose weight rapidly. A further analysis involving the use of detailed food diaries, along with possible body composition analysis, would allow more detailed conclusions to be drawn and should form the basis of future studies.

More than half (60%) of the patients had symptomatic dysphagia at the time of diagnosis. Increasing dysphagia scores were associated with increased total weight loss, increased rate of weight loss and reduced levels of food intake. Patients with gastro-oesophageal malignancy are not only subjected to the systemic effects of the disease on their nutritional status (for example, anorexia, hypermetabolism and altered protein metabolism), but also affected by the local effects of the tumour on the upper digestive tract. However, patients without dysphagia (and who had a ‘normal’ level of food intake) had still lost a median 4.4% of their total body weight by the time of diagnosis (rate, 1.5% per month) and therefore the systemic influences of the cancer on nutritional status remain important among these patients.

Stage of disease was also identified as an important determinant of rate of weight loss on multivariate analysis. Advanced disease stage may represent a more aggressive tumour biological behaviour with higher metabolic demands and a propensity for increased cell turnover and energy consumption. In addition, advanced disease stage may act as an indirect measure of tumour burden, which may not only place increased metabolic demands on the host, but may also result in increased production and release of biological mediators, such as pro-inflammatory cytokines and other tumour-derived products, that may contribute to the altered metabolism that is associated with the syndrome of cachexia. Increased size of the primary tumour has been linked with elevated serum CRP concentrations in patients with operable colorectal cancer, suggesting an association between tumour bulk and the magnitude of the systemic inflammatory response (Crozier *et al*, 2007).

Serum acute-phase protein concentrations correlated with total weight loss and rate of weight loss. Earlier, a similar association between elevated serum CRP concentrations and increased weight loss in pancreatic cancer patients has been identified and other groups have found similar associations in other cancer types (Falconer *et al*, 1995; O'Gorman *et al*, 1999; Barber *et al*, 1999a; Scott *et al*, 2002). In addition, REE in cancer patients is increased compared with controls and those patients with an APPR have greater REE compared with cancer patients without an APPR (Falconer *et al*, 1994). Cancer patients with an APPR have been shown to have elevated fibrinogen synthesis rates in both the fed and fasting states, and it is this reprioritisation of protein metabolism that accompanies the APPR that may also contribute to the wasting observed in cancer cachexia (Preston *et al*, 1998; Barber *et al*, 2000). In this study, CRP and albumin concentrations were identified as the best predictors of rate of weight loss by ROC curves. However, serum albumin concentrations may be influenced by malnutrition, hydration status and trans-capillary escape, as well as the presence of an inflammatory response (Fleck *et al*, 1985), and therefore serum CRP concentrations were chosen for entry into the multivariate analysis.

Serum cytokine concentrations were not measured in this study. The difficulties in measuring reliably circulating cytokine concentrations have been extensively documented elsewhere, and the relevance of such measurements must also be considered. Local tissue cytokine production by inflammatory cells (or tumour cells) is likely to be a better indicator of cytokine activity in cancer cachexia (Falconer *et al*, 1994; Martignoni *et al*, 2005). However, circulating acute-phase proteins (for example, CRP) remain robust indices of systemic pro-inflammatory status.

The findings of this study suggest that processes other than reduced food intake or mechanical obstruction alone are involved in the nutritional decline in patients with gastro-oesophageal malignancy. The realisation that weight loss in these patients may be composed of more than one component can modify therapeutic intervention. Recognition of the potential interactions between systemic inflammation and cancer may inform the development of appropriate therapeutic strategies to ameliorate weight loss, making patients more tolerant to cancer-modifying therapies such as surgery or chemotherapy (Fearon, 2008). It is also important to consider that the relative importance of these components may change during the course of an individual patient's illness, highlighting the need for periodic re-assessment and flexibility in terms of nutritional and therapeutic intervention. In addition, accelerated weight loss may provide one of the mechanisms by which systemic inflammation acts to reduce survival duration in a variety of different types of cancer (Falconer *et al*, 1995; Scott *et al*, 2002; Lamb *et al*, 2006; Deans *et al*, 2007).

## Figures and Tables

**Table 1:** Patient demographics and nutritional variables at the time of diagnosis (*n*=220)

	**Number (%)**
Age (years)^a^	71 (62–78)
*Sex*	
Male	145 (66)
Female	75 (34)
	
*Karnofsky score*	
30	4 (2)^b^
40	1 (1)
50	5 (2)
60	17 (8)
70	25 (12)
80	36 (17)
90	50 (24)
100	70 (34)
Unknown	12
	
*Tumour site*	
Oesophageal	101 (46)
Proximal third	2
Middle third	13
Distal third	86
Oesophago-gastric junction	40 (18)
Gastric	79 (36)
Proximal	25
Body	26
Distal	28
	
*Histology*	
Adenocarcinoma	185 (84)
Squamous cell carcinoma	30 (14)
Small cell	2 (1)
Indeterminate	3 (1)
	
*Grade*	
Well differentiated	3 (2)^b^
Moderately differentiated	63 (34)
Poorly differentiated	118 (64)
Not commented	36
	
*Subsequent treatment undertaken*	
Surgery alone	70 (32)
Pre-operative chemotherapy/surgery	25 (11)
Chemoradiotherapy with curative intent	7 (3)
Palliative chemotherapy	28 (13)
Palliative radiotherapy	6 (3)
Stent/dilatation/laser/symptomatic	84 (38)
	
*UICC stage*	
1	25 (11)
2	34 (16)
3	86 (39)
4	75 (34)
	
*Status*	
Alive	73 (33)
Dead	147 (67)
	
Pre-illness BMI	26.4 (24.1–30.1)
BMI at diagnosis	24.6 (21.4–28.0)
Total body weight loss (%)	7.1 (1.2–14.2)
Rate of weight loss (% per month)	2.5 (0.3–6.5)
	
*Dietary intake*	
Normal	85 (39)
Reduced	103 (47)
Poor/minimal	32 (14)
	
*Food diary intake* c	
Energy kcal (% of EAR)	87 (68–93)
Protein (% of RNI)	142 (109–170)
*Dysphagia score*	
0	89 (40)
1	52 (24)
2	43 (19)
3	32 (15)
4	4 (2)

BMI=body mass index; EAR=estimated average requirement; RNI=reference nutritional intake (%).

a Values are medians (inter-quartile range).

b Values are expressed as percentages of known results.

c Calculated from a subgroup of 22 patients.

**Table 2:** Association between rate of weight loss, clinicopathological variables and serum CRP concentrations (*χ*^2^ analysis)

	**Rate of weight loss (% body weight lost per month)**			
	**Lowest tertile**	**Mid tertile**	**Highest tertile**	***P*-value**
Age^a^	70 (64–77)	72 (62–77)	71 (62–78)	0.961
*Sex*				
M	47	52	46	0.419
F	26	21	28	
				
*Tumour site*				
Oesophageal	36	30	35	0.320
OGJ	8	18	13	
Gastric	29	25	27	
				
*Stage of disease*				
I	15	7	3	<0.001
II	21	9	3	
III	20	31	37	
IV	17	26	31	
				
*Treatment modality*				
Surgery alone	40	19	7	<0.001
Pre-operative chemotherapy and surgery	8	8	8	
Chemoradiotherapy	8	16	17	
Palliation	17	30	42	
				
*CRP (mg l*^−*1*^)				
<5	45	33	21	<0.001
⩾5	28	40	53	
				
*Dietary intake*				
Normal	49	23	14	<0.001
Reduced	21	39	42	
Poor/minimal	3	11	18	
				
*Dysphagia score*				
0	40	27	23	<0.001
1	23	16	11	
2	7	15	21	
3	2	13	17	
4	0	2	2	

CRP=C-reactive protein.

a Median (inter-quartile range); Kruskal–Wallis test.

**Table 3:** Multiple regression analysis of variables associated with increased weight loss

	**Regression coefficient**	**95% confidence interval**	**F-test**	**Estimates of effect size (%)**	***P*-value**
Dietary intake	0.28	0.87–2.44	1.9	38	<0.001
Stage of disease	0.19	0.24–1.34	0.9	28	0.003
CRP concentration (<5 or ⩾5 mg l^−1^)	0.18	0.40–2.54	1.3	34	0.007

CRP=C-reactive protein.

Dietary intake, stage of disease and serum CRP concentrations all retained independent associations in determining degree of weight loss. A multivariate general linear model was then used to calculate the estimates of size of effect on degree of weight loss for each variable.

## References

[bib1] Alexandrakis MG, Passam FH, Ganotakis ES, Sfiridaki K, Xilouri I, Perisinakis K (2003) The clinical and prognostic significance of erythrocyte sedimentation rate, serum inteleukin-6 and acute phase protein levels in multiple myeloma. Clin Lab Haematol 25(1): 41–46 12542441 10.1046/j.1365-2257.2003.00492.x

[bib2] Argiles JM, Alvarez B, Lopez-Soriano FJ (1997) The metabolic basis of cancer cachexia. Med Res Rev 17(5): 477–498 9276862 10.1002/(sici)1098-1128(199709)17:5<477::aid-med3>3.0.co;2-r

[bib3] Baracos V, Rodemann HP, Dinarello CA, Goldberg AL (2003) Stimulation of muscle protein degradation and prostaglandin E2 release by leukocytic pyrogen (interleukin-1). A mechanism for the increased degradation of muscle proteins during fever. N Engl J Med 308(10): 553–558 10.1056/NEJM198303103081002 6402699

[bib4] Barber MD, Fearon KCH, McMillan DC, Slater C, Ross JA, Preston T (2000) Liver export protein synthetic rates are increased by oral meal feeding in weight-losing cancer patients. Am J Physiol Endocrinol Metab 279(3): E707–E714 10950840 10.1152/ajpendo.2000.279.3.E707

[bib5] Barber MD, Powell JJ, Lynch SF, Gough NJ, Fearon KCH, Ross JA (1999a) Two polymorphisms of the tumour necrosis factor gene do not influence survival in pancreatic cancer. Clin Exp Immunol 117: 425–429a 10469042 10.1046/j.1365-2249.1999.01005.x PMC1905377

[bib6] Barber MD, Ross JA, Fearon KCH (1999b) Changes in nutritional, functional, and inflammatory markers in advanced pancreatic cancer. Nutr Cancer 35(2): 106–110 10693162 10.1207/S15327914NC352_2

[bib7] Baumann H, Gauldie J (1994) The acute phase response. Immunol Today 15: 74–80 7512342 10.1016/0167-5699(94)90137-6

[bib8] Bingham S, Wiggins HS, Englyst H, Seppanen R, Helms P, Strand R (1982) Methods and validity of dietary assessments in four Scandinavian populations. Nutr Cancer 4: 23–33 6296795 10.1080/01635588209513735

[bib9] Chen Z, Malhotra PS, Thomas GR, Ondrey FG, Duffey DC, Smith CW (1999) Expression of proinflammatory and proangiogenic cytokines in patients with head and neck cancer. Clin Cancer Res 5(6): 1369–1379 10389921

[bib10] Crozier JE, McMillan DC, McArdle CS, Angerson WJ, Anderson JH, Horgan PG, McKee RF (2007) Tumor size is associated with the systemic inflammatory response but not survival in patients with primary operable colorectal cancer. J Gastroenterol Hepatol 22(12): 2288–2291 18031394 10.1111/j.1440-1746.2006.04792.x

[bib11] Deans DAC, Wigmore SJ, de Beaux AC, Paterson-Brown S, Garden OJ, Fearon KCH (2007) Clinical prognostic scoring system to aid decision-making in gastro-oesophageal cancer. BJS 94: 1501–1509 10.1002/bjs.5849 17703501

[bib12] Department of Health (1991) Dietary reference values for food, energy and nutrients for the United Kingdom. 41. HMSO 1961974

[bib13] DeWys WD, Begg C, Lavin PT, Band PR, Bennett JM, Bertino JR (1980) Prognostic effect of weight loss prior to chemotherapy in cancer patients. Am J Med 69(4): 491–497 7424938 10.1016/s0149-2918(05)80001-3

[bib14] Falconer JS, Fearon KCH, Plester CE, Ross JA, Carter DC (1994) Cytokines, the acute phase response, and resting energy expenditure in cachectic patients with pancreatic cancer. Ann Surg 219(4): 325–331 7512810 10.1097/00000658-199404000-00001 PMC1243147

[bib15] Falconer JS, Fearon KCH, Ross JA, Elton R, Wigmore SJ, Garden OJ, Carter DC (1995) Acute phase protein response and survival duration of patients with pancreatic cancer. Cancer 75(8): 2077–2082 7535184 10.1002/1097-0142(19950415)75:8<2077::aid-cncr2820750808>3.0.co;2-9

[bib16] Fearon KC (2008) Cancer cachexia: Developing multimodal therapy for a multidimensional problem. Eur J Cancer 44(8): 1124–1132 18375115 10.1016/j.ejca.2008.02.033

[bib17] Fearon KCH, von Meyenfeldt MF, Moses AG, van Greenan R, Roy A, Gouma DJ (2003) Effect of a protein and energy dense N-3 fatty acid enriched oral supplement on loss of weight and lean tissue in cancer cachexia: a randomised double blind trial. Gut 52(10): 1479–1486 12970142 10.1136/gut.52.10.1479 PMC1773823

[bib18] Fleck A, Raines G, Hawker F, Trotter J, Wallace PI, Ledingham IM (1985) Increased vascular permeability: a major cause of hypoalbuminaemia in disease and injury. Lancet 1: 781–784 2858667 10.1016/s0140-6736(85)91447-3

[bib19] Gabay C, Kushner I (1999) Acute phase proteins and other systemic responses to inflammation. N Engl J Med 340(6): 448–454 9971870 10.1056/NEJM199902113400607

[bib20] Galizia G, Lieto E, De Vita F, Romano C, Orditura M, Castellano P (2002) Circulating levels of interleukin-10 and interleukin-6 in gastric and colon cancer patients before and after surgery: relationship with radicality and outcome. J Interferon Cytokine Res 22(4): 473–482 12034030 10.1089/10799900252952262

[bib21] Gelin J, Moldawer LL, Lonnroth C, Sherry B, Chizzonite R, Lundholm K (1991) Role of endogenous TNF*α* and IL-1 for experimental tumour growth and the development of cancer cachexia. Cancer Res 51(1): 415–421 1703040

[bib22] Gibson R (1990) Principles of Nutritional Assessment. Oxford University Press: Oxford

[bib23] Inagaki J, Rodriguez V, Bodey GP (1974) Causes of death in cancer patients. Cancer 33: 568–573 4591273 10.1002/1097-0142(197402)33:2<568::aid-cncr2820330236>3.0.co;2-2

[bib24] Kelson DP, Ginsberg R, Pajak T, Sheahan DG, Gunderson L, Mortimer J (1998) Chemotherapy followed by surgery compared with surgery alone for localised oesophageal cancer. N Engl J Med 339: 1979–1984 9869669 10.1056/NEJM199812313392704

[bib25] Knyrim K, Wagner H, Bethge N, Keymling M, Vakil N (1993) A controlled trial of an expansile metal stent for palliation of esophageal obstruction due to inoperable cancer. N Engl J Med 329(18): 1302–1307 7692297 10.1056/NEJM199310283291803

[bib26] Lamb GW, McMillan DC, Ramsey S, Aitchison M (2006) The relationship between the preoperative systemic inflammatory response and cancer-specific survival in patients undergoing potentially curative resection for renal clear cell cancer. Br J Cancer 94(6): 781–784 16523196 10.1038/sj.bjc.6603034 PMC3216422

[bib27] Martignoni ME, Kunze P, Hildebrandt W, Kunzli B, Berberat P, Giese T (2005) Role of mononuclear cells and inflammatory cytokines in pancreatic cancer-related cachexia. Clin Cancer Res 11: 5802–5808 16115919 10.1158/1078-0432.CCR-05-0185

[bib28] McKernan M, McMillan DC, Anderson JR, Angerson WJ, Stuart RC (2008) The relationship between quality of life (EORTC QLQ-C30) and survival in patients with gastro-oesophageal cancer. Br J Cancer 98(5): 888–893 18268490 10.1038/sj.bjc.6604248 PMC2266859

[bib29] O'Gorman P, McMillan DC, McArdle CS (1999) Longitudinal study of weight, appetite, performance status and inflammation in advanced gastrointestinal cancer. Nutr Cancer 35(2): 127–130 10693165 10.1207/S15327914NC352_5

[bib30] Preston T, Slater C, McMillan DC, Falconer JS, Shenkin A, Fearon KC (1998) Fibrinogen synthesis is elevated in fasting cancer patients with an acute phase response. J Nutr 128(8): 1355–1360 9687556 10.1093/jn/128.8.1355

[bib31] Ross PJ, Ashley S, Norton A, Priest K, Waters JS, Eisen T, Smith IE, O'Brien ME (2004) Do patients with weight loss have a worse outcome when undergoing chemotherapy for lung cancers? Br J Cancer 90(10): 1905–1911 15138470 10.1038/sj.bjc.6601781 PMC2409471

[bib32] Scott HR, McMillan DC, Forrest LM, Brown DJ, McArdle CS, Milroy R (2002) The systemic inflammatory response, weight loss, performance status and survival in patients with inoperable non-small cell lung cancer. Br J Cancer 87(3): 264–267 12177792 10.1038/sj.bjc.6600466 PMC2364225

[bib33] Selby P, Hobbs S, Viner C, Jackson E, Jones A, Newall D (1987) Tumour necrosis factor in man: clinical and biological observations. Br J Cancer 56: 803–808 3435706 10.1038/bjc.1987.294 PMC2002406

[bib34] Stouthard JML, Romijn JA, van der Poll T, Endert E, Klein S, Bakker PJM (1995) Endocrinologic and metabolic effects of interleukin-6 in humans. Am J Physiol 268: E813–E819 7762632 10.1152/ajpendo.1995.268.5.E813

[bib35] Strassmann G, Fong M, Kenney JS, Jacob CO (1992) Evidence for the involvement of interleukin 6 in experimental cancer cachexia. J Clin Invest 89(5): 1681–1684 1569207 10.1172/JCI115767 PMC443047

[bib36] Studley HD (1936) Percentage of weight loss. A basic indicator of surgical risk in patients with chronic peptic ulcer disease. JAMA 106: 458–460 11680474

[bib37] Tamakoshi K, Yatsuya H, Kondo T, Hirano T, Hori Y, Yoshida T, Toyoshima H (2003) The accuracy of long-term recall of past body weight in Japanese adult men. Int J Obesity 27: 247–252 10.1038/sj.ijo.802195 12587006

[bib38] Wigmore SJ, Fearon KCH, Lai PBS, O'Riordain MG, Falconer JS, Maingay JP, Ross JA (1997) Recombinant and tumour-cell derived IL-8 stimulates acute phase protein production by isolated human hepatocytes. Am J Physiol 273: E720–E726 9357801 10.1152/ajpendo.1997.273.4.E720

[bib39] Wigmore SJ, Fearon KCH, Sangster K, Maingay JP, Garden OJ, Ross JA (2002) Cytokine regulation of constitutive production of IL-8 and –6 by human pancreatic cancer cell lines and serum cytokine concentrations in patients with pancreatic cancer. Int J Oncol 21(4): 881–886 12239630 10.3892/ijo.21.4.881

